# From Waste to Worth: The Role of Fermentation in a Sustainable Future

**DOI:** 10.3390/foods15040664

**Published:** 2026-02-12

**Authors:** Morena Gabriele, Laryssa Peres Fabbri, Maria Ventimiglia, Anna Łepecka

**Affiliations:** 1 Institute of Agricultural Biology and Biotechnology, National Research Council (IBBA-CNR), 56124 Pisa, Italy; morena.gabriele@ibba.cnr.it (M.G.); laryssaperesfabbri@cnr.it (L.P.F.); maria.ventimiglia@ibba.cnr.it (M.V.); 2 Institute of Agricultural and Food Biotechnology—State Research Institute, 02532 Warsaw, Poland

**Keywords:** fermentation biotechnology, food waste valorization, circular bioeconomy, gut microbiome and immunity, postbiotics, human health, precision fermentation, sustainable food systems, bioactive compounds recovery

## Abstract

Fermentation, one of the oldest biotransformation processes, has become a key element of contemporary sustainable biotechnology. In modern food systems, it enables the simultaneous resolution of environmental, nutritional, and economic challenges by converting agricultural and food residues into high-value-added products, such as bioactive compounds, organic acids, biofuels, enzymes, and proteins. Consistent with the concept of a circular bioeconomy, fermentation supports resource recycling, waste minimization, and greenhouse gas reduction, contributing to the achievement of selected United Nations Sustainable Development Goals (SDGs). The importance of fermentation extends beyond its environmental aspects—fermented foods and postbiotics support the modulation of the gut microbiome, strengthen immunity, and can act as a preventative measure against metabolic and inflammatory conditions. Simultaneously, the dynamic development of precision fermentation and synthetic biology enables the design of microorganisms that produce specific food ingredients without the use of animals or traditional agriculture, paving the way for more responsible production and consumption. This review presents the categories of organic residues valorized through fermentation, explains their role in circular food and healthcare systems, and identifies key technological and regulatory barriers limiting the scaling of this approach. Collectively, fermentation emerges as a biotechnology platform with significant transformative potential for future sustainable food systems.

## 1. Introduction—Sustainability and the Challenges of Contemporary Food Systems

Contemporary food systems are under growing pressure to meet the nutritional needs of a rising global population while operating within the planet’s ecological boundaries. They face multiple, interlinked sustainability challenges, including the generation of massive volumes of edible and inedible food waste, the inefficient use of natural resources (land, water, energy, and nutrients), and the increasing contribution to greenhouse gas (GHG) emissions, biodiversity loss, and soil degradation [1].

The global food system accounts for approximately one-third of anthropogenic GHG emissions and consumes over 70% of freshwater resources, largely driven by land-based activities such as agriculture and land-use change [2,3,4]. Simultaneously, an estimated 1.3 billion tonnes of food is lost or wasted each year—approximately one-third of total production—resulting in significant losses of natural resources and a substantial climate burden [5,6,7].

Addressing these systemic inefficiencies is essential to achieving the United Nations Sustainable Development Goals (SDGs), particularly SDG 2 (“Zero Hunger”), SDG 3 (“Good Health and Well-being”), SDG 7 (“Affordable and Clean Energy”), SDG 12 (“Responsible Consumption and Production”), and SDG 13 (“Climate Action”) [8]. The transition toward sustainability requires not only technological innovation but also the restructuring of production and consumption systems, underpinned by circular bioeconomy principles that prioritize resource recirculation, waste minimization, and value retention throughout the food chain [9].

Growing attention is being directed toward the recovery and recycling of food waste, as these processes can simultaneously mitigate GHG emissions and generate renewable energy and valuable resources. Such integrated valorization approaches offer opportunities for both immediate environmental benefits and long-term economic returns [10]. Among the various bio-based solutions, fermentation has emerged as a particularly flexible and scalable approach. Major sources of fermentation substrates originate from specific food-industry sectors, including fruit and vegetable processing (rich in carbohydrates and polyphenols), cereal milling and brewing (starch- and fiber-rich residues), dairy processing (whey- and lactose-rich streams), and meat and fish processing (protein- and lipid-rich by-products) [11,12,13,14]. The physicochemical characteristics of these streams strongly influence fermentation performance and product yield.

Unlike thermochemical conversions, microbial fermentation operates under mild conditions and selectively transforms complex organic residues into nutritionally valuable or industrially relevant products, such as organic acids, bioactive compounds, proteins, and biopolymers 5,10]. At the same time, fermentation’s biological and nutritional outcomes align closely with health-oriented sustainability objectives. Fermentation improves the digestibility, safety, and functional quality of several food products [15]. Fermented foods can modulate the gut microbiome in both the short and long term, influence immune responses, and contribute to metabolic health and disease prevention [16,17].

Furthermore, the emergence of precision fermentation, driven by synthetic biology and metabolic engineering, is redefining the potential of microbial biotechnology. Precision fermentation employs engineered microorganisms to produce targeted proteins, lipids, enzymes, or bioactive compounds, thereby reducing land use, GHG emissions, and reliance on livestock or resource-intensive crops. These innovations expand fermentation’s role beyond waste valorization to include sustainable ingredient design, alternative protein production, and circular bio-manufacturing [18,19]. Harnessing microbial biosynthetic capacities through precision fermentation enables the efficient, large-scale, and cost-effective production of a broad range of targeted compounds, meeting the growing demand for sustainable ingredients in both the food and chemical sectors [18]. Finally, integrating fermentation within the circular bioeconomy provides a systemic response to global food-system challenges. By converting agricultural residues, processing by-products, and post-consumer waste into new resources, fermentation closes material loops, mitigates pollution, and generates added value at multiple stages of the supply chain [19,20].

In summary, fermentation stands at the intersection of environmental sustainability, health, and technological innovation, serving both as a regenerative pathway for the valorization of bioeconomy side streams and as a platform for producing nutritious, functional, and low-impact foods. This review adopts an integrated perspective that connects fermentation-based waste valorization with sustainability goals, circular bioeconomy principles, human health implications, regulatory considerations, and emerging technologies such as precision fermentation. While previous studies have often focused on individual product categories or specific aspects of food waste fermentation, this work aims to bridge these domains within a unified framework. The manuscript is structured as follows: Section 2 discusses the contribution of fermentation to the Sustainable Development Goals; Section 3, Section 4 and Section 5 describe fermentation principles and major valorization pathways; Section 6 addresses precision fermentation; Section 7 examines microbiome and health-related aspects; Section 8 addresses postbiotics and sustainable development, and Section 9 and Section 10 critically discuss challenges, limitations, and future perspectives.

## 2. Fermentation and Sustainable Development Goals (SDGs)

Fermentation technologies play a pivotal role in advancing the United Nations SDGs by bridging microbial biotechnology with the transformation of global food systems. Beyond general linkages, specific SDG targets are influenced by fermentation through measurable indicators in the UN SDG indicator [21] framework. For example:

- Indicator 2.1.1 (Prevalence of undernourishment) is relevant to fermentation contributions to alternative protein sources;
- Indicator 2.3.1 (Volume of production per labour unit by classes of farming/pastoral/forestry enterprise size) can be influenced by fermentation-enhanced biostimulants and biofertilizers;
- Indicator 12.5.1 (National recycling rate, tons of material recycled) reflects fermentation’s role in valorizing bioeconomy side streams;
- Indicator 7.2.1 (Renewable energy share in total final energy consumption) captures biofuels and biogas production through fermentation;
- Indicator 13.2.1 (Number of countries that have communicated the establishment or operationalization of an integrated policy/strategy/plan which increases their ability to adapt to the adverse impacts of climate change, and foster climate resilience and low greenhouse gas emissions development in a manner that does not threaten food production (including a national adaptation plan, nationally determined contribution, national communication, biennial update report or other)) aligns with fermentation-based climate resilience technologies.

**Figure 1:**
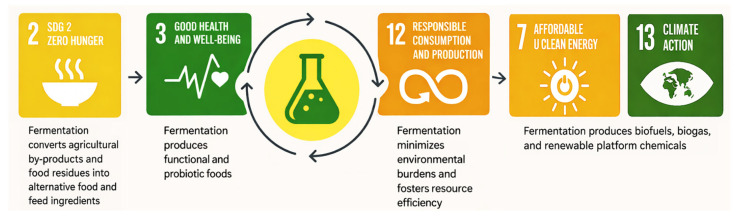
Fermentation and Sustainable Development Goals.

Through its diverse applications—from waste valorization and bioenergy generation to nutritional improvement—fermentation contributes to multiple interrelated SDG targets (Figure 1). As illustrated in Figure 1, fermentation-based processes contribute to multiple interconnected SDGs by simultaneously addressing food quality and security, waste reduction, climate mitigation, and health promotion. The figure highlights these linkages, emphasizing that the sustainability impact of fermentation arises not from a single application but from the integration of resource valorization, nutritional enhancement, and environmental benefits across the food system.

SDG 2 (Zero Hunger) benefits from fermentation’s ability to convert agricultural by-products and food residues into alternative ingredients. These products can be broadly distinguished into food and feed applications and non-food applications, with demonstrable contributions to SDG indicators: Indicator 2.1.1 (prevalence of undernourishment) and Indicator 2.3.1 (agricultural productivity). Food- and feed-oriented fermentation products include single-cell proteins (SCP) and other bioactive microbial ingredients, which directly contribute to human and animal nutrition by providing alternative protein sources and functional compounds [10]. These processes enhance food security and reduce pressure on cropland and traditional animal-based proteins. In contrast, non-food fermentation products such as biofertilizers and industrial enzymes do not directly enter the food chain but play a critical supporting role in sustainable agriculture, influencing productivity (e.g., Indicator 2.3.1) and soil health. Fermentation-derived biofertilizers and soil amendments improve nutrient availability, soil fertility, and crop performance, thereby indirectly supporting sustainable agricultural production [22,23]. Similarly, industrial enzymes produced via microbial fermentation are widely used in food processing, feed production, and biobased industries, increasing process efficiency, reducing resource inputs, and lowering environmental impacts [24].

SDG 3 (Good Health and Well-being) is supported by fermentation that promotes the production of functional foods, probiotics, and nutrient-enhanced foods that improve gut and immune health while mitigating the risks of metabolic and chronic diseases [16,17]. Fermented foods often exhibit enhanced nutritional and health-promoting properties due to microbial transformation and bioactive compound generation [15]. In this sense, fermentation acts as a biological bridge between sustainable production and preventive health, supporting both environmental and nutritional resilience.

Through SDG 12 (Responsible Consumption and Production), fermentation embodies the principles of the circular bioeconomy. By converting agricultural by-products, food-processing residues, and post-consumer waste into value-added products instead of discarding them, fermentation supports Indicator 12.5.1 on recycling and reuse, minimizes environmental burdens and fosters responsible resource management [10,25,26]. The integration of fermentation helps close nutrient loops, reduce waste, and support sustainable material flow. This is reinforced by studies showing microorganisms are directly or indirectly relevant to every SDG target, especially in the context of sustainable agriculture, nutrition, and health [27].

SDG 7 (Affordable and Clean Energy) and SDG 13 (Climate Action) are supported by fermentation through the generation of biofuels, biogas, and renewable platform chemicals from organic residues, thereby contributing to Indicator 7.2.1, Indicator 13.2.1, and broader mitigation efforts by replacing fossil-based processes and contributing to climate mitigation [25,26]. Large-scale integration of biobased fermentation is viewed as essential for a carbon-neutral circular economy by 2050, with diverse fermentation platforms expected to supply the bulk bio-based chemicals needed to replace fossil-derived products [25].

Beyond individual goals, fermentation exemplifies the interconnected nature of the SDGs. It simultaneously addresses challenges in food security, public health, waste reduction, and climate change, illustrating the synergies that can emerge from integrated microbial and circular-bioeconomy strategies. Ultimately, fermentation is not merely a food-processing tool or waste-management technology, but a strategic enabler of sustainable development. Indeed, by promoting resource efficiency, reducing waste and emissions, supporting health, and fostering inclusive bioeconomic growth, fermentation offers a pathway toward resilient and low-impact food systems across SDG targets.

In addition to food, feed, and industrial applications, the fermentation of bioeconomy side streams is increasingly recognized as a promising route for the production of plant biostimulants. Plant biostimulants encompass a broad range of substances and microorganisms that, when applied to plants or soils, enhance plant growth, nutrient uptake, and resilience to climate-induced abiotic stresses, thereby improving crop performance beyond what can be achieved by conventional fertilization alone [28]. These effects address Indicator 2.3.1 by potentially increasing crop yields and stability under stress conditions.

A major class of fermentation-derived biostimulants is represented by protein hydrolysates, complex mixtures of peptides and free amino acids produced by enzymatic or chemical breakdown of proteins from agro-industrial by-products (e.g., crop residues, animal processing side streams) [29]. These hydrolysates have been shown to stimulate plant physiological processes, including nutrient assimilation, carbon and nitrogen metabolism, root architecture development, and hormone-like signaling, resulting in improved nutrient use efficiency and biomass accumulation across horticultural and agronomic crops [29]. Moreover, protein hydrolysates have been reported to mitigate abiotic stresses such as drought, salinity, and temperature extremes by enhancing antioxidant capacity and osmotic adjustment mechanisms, contributing to resilience under climatic extremes [30].

Beyond protein hydrolysates, microbial metabolites (e.g., organic acids and phytohormone-like compounds) and microorganisms themselves, such as plant-growth-promoting rhizobacteria (PGPR) and beneficial fungi, are increasingly recognized as biostimulants that enhance nutrient solubilization, root growth, water use efficiency, and stress tolerance through multiple biochemical and ecological pathways [31]. Collectively, these fermentation-derived biostimulants contribute to climate-resilient agriculture by improving crop productivity while reducing dependence on synthetic fertilizers and agrochemicals. This approach directly supports SDG 2 (Zero Hunger) through enhanced and more stable agricultural yields and SDG 13 (Climate Action) by lowering environmental impacts and increasing crop resilience under changing climatic conditions [32]. Crucially, the use of agro-industrial residues as substrates for biostimulant production reinforces circular bioeconomy principles by closing nutrient loops and valorizing low-value biomass streams that would otherwise be discarded.

## 3. Definition and Types of Fermentation, the Importance of the Fermenta-Tion Process

### 3.1. Definition and Functional Role of Fermentation

Fermentation is a controlled microbial process in which microorganisms—primarily bacteria, yeasts, and filamentous fungi—catabolise organic substrates such as carbohydrates, proteins, and organic acids. Biochemically, it is characterised by an internal redox balance, with organic compounds acting as both electron donors and acceptors [33,34,35,36], producing end-products such as lactic acid, ethanol, and organic acids, along with gases such as carbon dioxide and various bioactive metabolites.

While fermentation is strictly defined as an anaerobic process, in the broader context of industrial bioprocessing, the term encompasses any large-scale microbial cultivation. Consequently, a fundamental distinction is made based on oxygen management, categorising these systems as either aerobic or anaerobic. This distinction is a critical driver of bioprocess kinetics: aerobic pathways generally allow for faster microbial growth and more efficient energy yields, leading to shorter processing cycles. Conversely, anaerobic processes, though often characterised by slower metabolic rates, are frequently easier to scale in industrial settings as they eliminate the high energy costs and technical complexities associated with constant aeration [35]. Regardless of the oxygen regime, the processing time remains a pivotal performance indicator; it dictates the overall productivity by defining the rate at which a specific yield (g/L) is achieved, thereby serving as a primary determinant for the economic feasibility and volumetric throughput of a biorefinery [10].

During fermentation, extensive enzymatic activity leads to the hydrolysis of polysaccharides and proteins into smaller molecules, improving digestibility and modifying physicochemical properties such as texture, acidity, and flavour [37]. The increased bioavailability of nutrients following fermentation is supported by several studies reporting enhanced assimilation of B-group vitamins, minerals, and amino acid nitrogen [38]. This nutrient-improving capacity contributes to the long-standing dietary relevance of fermented foods, supporting their increasing incorporation into contemporary dietary guidelines that focus on gut health and functional nutrition.

### 3.2. Major Types of Fermentation

Fermentation processes can be classified based on two main criteria: the primary metabolic products generated or the technological configuration of the production system. Such classifications provide a framework to understand the diverse applications of fermentation, spanning food production, industrial biotechnology, and biochemical manufacturing.

#### 3.2.1. Classification by Metabolic Pathway

Lactic acid fermentation, carried out by lactic acid bacteria (LAB) such as *Lactobacillus* and *Streptococcus*, results in the accumulation of lactic acid, the lowering of pH, and the inhibition of spoilage microorganisms. It is central to the production of yoghurt, cheese, fermented vegetables, cured meats, and non-dairy functional foods, contributing to flavour development, nutrient availability, and microbial stability [39,40]. From an industrial perspective, maintaining high acidification rates while mitigating the risk of bacteriophage contamination remains a primary operational challenge to ensure batch consistency.

Alcoholic fermentation, primarily performed by *Saccharomyces cerevisiae*, converts fermentable sugars into ethanol and carbon dioxide, supporting the production of beer, wine, sake, and leavened bakery products [41,42,43]. However, achieving maximum theoretical yields is often constrained by ethanol-induced toxicity and the metabolic stress faced by yeasts in high-gravity substrates.

Propionic acid fermentation, used for Swiss-type cheese and organic acid production, produces propionate, acetate, and vitamin B_12_. Recent advances focus on metabolic engineering and substrate optimization to improve yields and overcome the slow growth kinetics typical of these pathways [44,45,46]. Other industrially relevant fermentations include butyric acid fermentation by *Clostridium* spp. and acetic acid oxidation by *Acetobacter*. While the former requires strict anaerobiosis, the latter is an aerobic process; both, however, present significant technological challenges related to oxygen transfer efficiency and product recovery costs [47,48].

#### 3.2.2. Classification by Production System Configuration

Industrial fermentation systems can be broadly divided into submerged fermentation (SmF) and solid-state fermentation (SSF), depending on the physical nature of the substrate and operational strategy. While both systems can be applied in food and biochemical production, their use is often context-dependent.

In SmF, microorganisms grow in a liquid medium that provides a homogeneous environment and supports efficient nutrient uptake. This system allows precise control of parameters such as pH, temperature, and oxygen availability, which is essential for consistent microbial growth and metabolite production. In aerobic SmF configurations, the ability to maintain high oxygen transfer rates can significantly reduce processing times, accelerating the production of target metabolites. However, the substantial energy demand for mechanical stirring and high-pressure air injection remains a major challenge for the scalability of these systems [49]. This operational burden exemplifies the technical complexities of aerobic regimes mentioned in the previous section. SmF is widely applied in industrial processes for enzymes, antibiotics, recombinant proteins, and organic acids [49,50]. It is also used in some food fermentations, particularly liquid-based products such as beverages.

Solid-state fermentation (SSF) relies on low–water activity solid substrates, such as cereal by-products, pulses, or agro-industrial residues, which function both as nutrient sources and physical support for microbial growth. Traditionally used in processes like East Asian “Koji,” SSF is now recognized for key biotechnological and environmental advantages: it enables high product yields while minimizing water demand, wastewater generation, and overall energy consumption compared with SmF [51,52,53,54]. This environmental advantage is further supported by a life-cycle assessment showing that cellulase production via SSF on coffee husk requires less energy and water than SmF using *Trichoderma reesei* [55]. Despite these benefits, SSF is frequently characterised by longer processing times compared to SmF, due to limited mass transfer and slower nutrient diffusion within the solid matrix. This extended duration represents a significant constraint for industrial scalability. In line with the productivity principles established in Section 3.1, the longer residence times required in the reactor to reach target metabolite concentrations (g/L) directly limit the total annual output of the facility [51].

SSF faces engineering challenges, particularly at large scale, due to heterogeneity in heat and mass transfer and difficulties in maintaining uniform moisture and oxygen profiles [56]. In industrial scale-up, these challenges are exemplified by the buildup of metabolic heat; limited heat removal and conduction through the solid matrix can lead to supra-optimal internal temperatures, as reviewed by Singhania et al. [57]. Experimental studies in pilot packed-bed SSF bioreactors have quantified heat and mass transfer coefficients and shown that temperature profiles in the bed are strongly influenced by airflow distribution and the lack of thermal equilibrium between air and solid phases, indicating that heat dissipation can be difficult to manage at larger scales [58]. Furthermore, as reviewed by Singhania et al. [57], the lack of effective agitation in large reactors creates barriers to oxygen transfer, necessitating complex aeration strategies to avoid anaerobic zones that reduce overall yield. To overcome these limitations, hybrid systems combining SSF and SmF, with substrate recycling between reactors, have been developed, improving treatment efficiency and reducing overall reactor volume [59]. The choice between SmF and SSF depends on microbial strain, substrate nature, target metabolite, and the industrial or food production context. As summarised in Table 1, several studies show that SSF can outperform SmF for enzyme production: for example, *Neurospora sitophila* yields higher cellulase titres in SSF on lignocellulosic substrates [60], while *Aspergillus niger* and *Aspergillus brasiliensis* also show higher enzyme production under SSF conditions [61,62]. In contrast, *Aspergillus oryzae* exhibits different growth and secretion profiles depending on the fermentation mode, highlighting the strain- and condition-specific nature of the comparison [63]. This variability is reflected in industrial practice, where certain bulk metabolites such as citric acid are predominantly produced via SmF due to better process control and volumetric productivity [64].

### 3.3. Strategic Importance in Food Systems and Human Nutrition

Fermentation is increasing in importance due to its technological versatility, reproducibility, and capacity to improve product stability and functionality. It contributes to microbiological safety by generating organic acids and other antimicrobial metabolites, lowering pH, and allowing beneficial microorganisms to outcompete undesirable ones. In addition, in several fermented food matrices, modifications in water availability further limit microbial growth [65,66]. These mechanisms explain the historical role of fermentation as a natural preservation technique and its continued relevance in regions without extensive cold-chain infrastructure.

In recent years, large-scale sequencing and metabolomics technologies have enabled a more detailed characterisation of the complex biochemical and microbial interactions occurring during fermentation, leading to improved process control and strain development. Multi-omics studies have been particularly effective in mapping metabolic pathways responsible for aroma development, proteolysis, acidification kinetics, and microbe–microbe interactions in multi-species fermentations [67,68].

As a result, fermentation represents both a deeply rooted food technology and an expanding industrial discipline that benefits from advances in microbial engineering, computational modelling, and analytical chemistry. Its historical relevance, mechanistic predictability, and alignment with consumer demand for minimally processed yet functionally enriched foods ensure its continued strategic role in shaping the future of food biotechnology.

## 4. Fermentation in the Circular Bioeconomy

The transition from a linear to a circular economic model represents a major challenge for the agrifood sector. Conventional linear systems, characterised by production, consumption, and disposal, lead to resource depletion, environmental pollution, and elevated GHG emissions.

Fermentation has become a central technology to support circularity by converting organic waste streams into valuable products while closing nutrient and carbon loops [25,69,70]. Integrating fermentation into biorefinery frameworks allows the agrifood sector to recover resources, generate bio-based chemicals, and reduce environmental burdens simultaneously. Globally, the agri-food sector generates massive quantities of organic residues, over 190 million tons annually, including fruit and vegetable by-products, dairy waste, spent grains, molasses, peels, husks, and crop stalks [71]. These materials are a rich source of compounds such as polyphenols, dietary fibers, and proteins that can be recovered and utilized in functional food systems [72].

In this scenario, microbial fermentation represents a strategy for valorising these resources. Microorganisms can degrade complex polymers, such as cellulose, hemicellulose, and proteins, and transform them into economically and industrially valuable compounds. This approach contributes to both reducing waste and generating high-value bio-products.

Fermentation processes enable the production of several high-value compounds, including platform chemicals like organic acids (e.g., succinic acid, lactic acid, and itaconic acid). For example, *Actinobacillus succinogenes* and *Corynebacterium glutamicum* efficiently convert lignocellulosic hydrolysates into succinic acid, a precursor for bioplastics and pharmaceuticals [73,74]. Another key output is SCP, microbial biomass derived from yeasts, fungi, or microalgae, which converts waste streams into protein-rich material suitable for feed or human consumption [75]. Furthermore, solid-state fermentation of fruit and vegetable residues can produce other bio-based molecules such as biosurfactants, exopolysaccharides, prebiotics, antimicrobials, pigments, and enzymes [76].

Advanced approaches, such as co-cultivation and metabolic engineering, are being developed to enhance yields, expand substrate ranges, and enable simultaneous production of multiple compounds, further increasing the circularity potential. Diverting organic waste from landfills, major sources of methane emissions, into controlled fermentation processes provides direct climate mitigation benefits.

Fermentation reduces dependence on fossil-derived chemical production. Life cycle assessment (LCA) studies indicate that fermentation-based valorization of food waste can decrease carbon footprints, lower water consumption, and minimize land use compared to conventional disposal or chemical synthesis [77,78,79]. Fermentation also facilitates nutrient recycling: nitrogen and phosphorus retained in microbial biomass can be reincorporated into agricultural systems, effectively closing nutrient loops. Integrating fermentation with downstream energy recovery, such as anaerobic digestion of residual streams, further enhances sustainability by producing renewable energy while minimizing waste.

**Figure 2:**
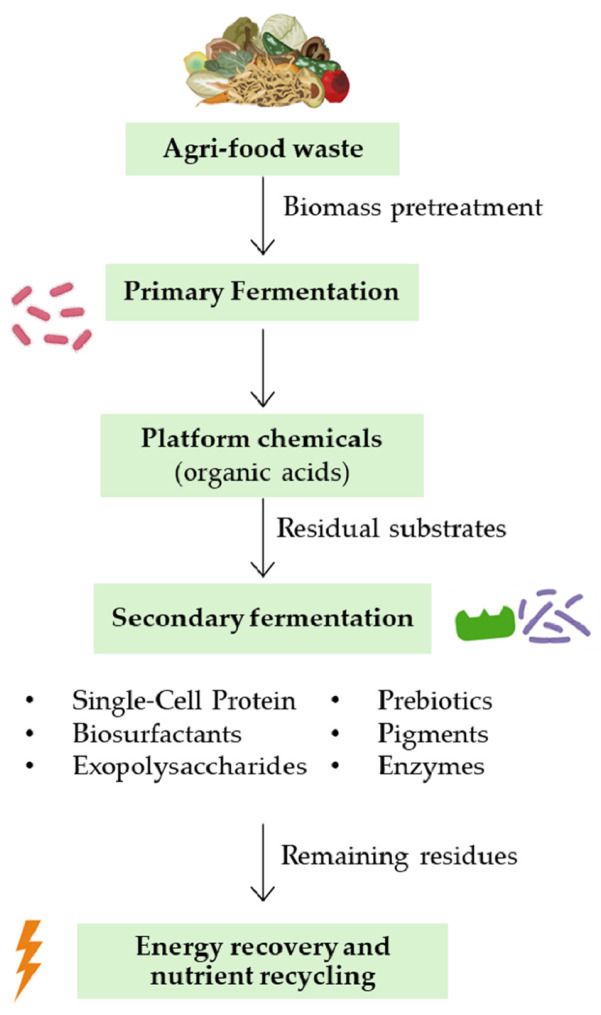
Schematic representation of the cascading fermentation–biorefinery model.

Within circular biorefinery architectures, fermentation functions as a biological interface connecting upstream biomass pretreatment to downstream product recovery. Cascading approaches maximize the valorization of all biomass fractions (Figure 2): primary fermentation converts pretreated biomass into platform chemicals for industrial applications [77,80]; residual streams then proceed to a secondary fermentation, serving as substrates for SCP or the production of other high-value bio-based molecules [81,82].

Finally, residual valorization and energy recovery are achieved by processing the remaining residues via anaerobic digestion for biogas production [83]. Even spent fermentation media can be recycled, supporting secondary fermentations and reducing overall process costs [84]. This multi-level approach improves economic feasibility and exemplifies the integration of biological, chemical, and energy valorization pathways within a circular system.

## 5. Valorization of Organic Waste Through Fermentation

Fermentation serves as a versatile strategy for the valorization of agri-food residues, enabling the production of various added-value compounds; these range from nutritional and functional molecules (e.g., proteins, enzymes, and bioactive compounds) to industrial products such as organic acids and biofuels.

### 5.1. Single-Cell Protein (SCP)

With the global population projected to reach 9.3 billion by 2050, the demand for sustainable protein is rising. Millions face undernourishment, while food waste remains high, highlighting the need for innovative solutions.

SCPs from yeasts, fungi, bacteria, or algae provide essential amino acids, vitamins, and minerals in a cost-effective, scalable, and environmentally friendly way [85]. Owing to microbial growth in bioreactors, SCP can achieve volumetric productivities far exceeding those of conventional plant- and animal-derived proteins, surpassing agricultural outputs by several orders of magnitude on a land-use basis [86]. Moreover, SCP production requires substantially less land and water, does not directly compete with food crops, and can valorize low-cost organic residues, thereby reducing greenhouse gas emissions and organic waste disposal [87]. Scenario analyses suggest that replacing 20% of global beef consumption with microbial protein could reduce cumulative deforestation by up to 50% by 2050 [88].

Compared with chemical extraction or thermochemical conversion processes, microbial fermentation operates under milder conditions and enables year-round, climate-resilient production. Nevertheless, energy demand associated with aeration, agitation, sterilization, and downstream processing remains a key limitation [89]

The use of fruit and agricultural residues as fermentation substrates represents a key strategy to enhance the sustainability of SCP processes, supporting circular bioeconomy principles by converting low-value organic waste into protein (Table 2).

The fermentation of fruit wastes, such as mango, prickly custard apple, pineapple, papaya, banana, mangosteen, cashew apple, cacao, jackfruit, and pomegranate, using *Saccharomyces cerevisiae*, represents a sustainable approach to protein production [90]. For example, pineapple residues can yield up to 3.01 kg/m^3^ of SCP at 60% substrate concentration, demonstrating strong potential for waste valorization [91].

Comparative studies at laboratory scale indicate that fungal SCP production from orange, banana, sugarcane, garlic, and potato peels can achieve competitive protein levels, with *Aspergillus niger* on pineapple waste reaching 9.79 g/L after 10 days, the highest reported among the examined wastes [92]. In other studies, submerged fermentation of potato peels for 7 days yielded the highest SCP among banana, citrus, and carrot residues [93]. Filamentous fungi have been employed to enhance the protein levels in different food processing residues, including potato starch wastewater and leftover bread [94,95]. At an industrial-scale trial, *Rhizopus delemar* efficiently converted potato-processing waste into fungal biomass containing 53% crude protein [95]. Moreover, coffee wastewater, rich in nutrients, can also be used for SCP production; for example, Pillaca-Pullo et al. [96] achieved 64.4% SCP yield using *Candida sorboxylosa* under optimized laboratory-scale conditions.

Despite these promising results, large-scale deployment of SCP production from organic residues remains constrained by several challenges, including substrate heterogeneity, the presence of inhibitory compounds, high nucleic acid content, and safety concerns related to toxins and allergenicity [87]. The safety of SCP products depends critically on substrate quality, microbial strain selection, and downstream processing strategies aimed at removing undesirable compounds. Moreover, compliance with feed and food regulatory frameworks—including requirements related to composition, contaminants, labeling, traceability, and the European Food Safety Authority’s (EFSA) Qualified Presumption of Safety (QPS) guidelines—is essential to ensure product safety and consumer acceptance.

Overall, SCP represents a highly scalable and low-carbon protein source with significant potential to contribute to sustainable food systems. However, its industrial viability depends on improvements in energy-efficient fermentation, cost-effective downstream processing, careful microbial and substrate selection, and strict adherence to regulatory standards. Integrating comprehensive sustainability assessments that encompass environmental, nutritional, and socio-economic metrics will be crucial to support the transition of SCP from laboratory-scale demonstrations to robust commercial applications.

### 5.2. Biofuels

Growing environmental concerns associated with fossil fuel consumption have accelerated the search for renewable alternatives such as wind, solar, and biomass-derived fuels [97]. Among these, bioethanol, 2,3-butanediol (BDO), and biohydrogen are key biofuels produced through microbial fermentation of carbohydrate-rich residues as shown in Table 3.

Bread waste, abundant in Europe, can be efficiently converted into BDO by *Enterobacter ludwigii*, achieving fed-batch yields of 135–145 g/L with conversion efficiencies of 0.42–0.48 g/g [98]. Bioethanol is particularly attractive due to its compatibility with existing fuel infrastructures and its production from lignocellulosic biomass, food waste, and agro-industrial by-products [98]. Second-generation ethanol can be obtained from kitchen waste using a co-culture of *Saccharomyces cerevisiae* and *Pichia stipitis*, under optimized pH and nitrogen conditions, achieving ethanol titers up to 45 g/L [100], while enzymatic hydrolysis by *Fusarium oxysporum* followed by co-cultivation with *S. cerevisiae* produced 30.3 g/L from food waste [99].

Several studies comparing different agricultural residues report that corn-processing by-products are among the most efficient substrates for butanol production by *Clostridium* spp. [101,102]. Low-cost biohydrogen production has also been demonstrated using non-sterile vegetable waste and indigenous microbial consortia, reaching up to 85.65 mL H_2_ per gram of volatile solids [104], while de-oiled rice bran hydrolysates fermented with *Clostridium acetobutylicum* YM1 yielded 572.5 mL of hydrogen (132.2 mL H_2_ per g sugars) [103]. In comparison, thermochemical routes such as gasification, pyrolysis, and steam reforming can achieve higher hydrogen yields (up to 200 mmol H_2_/g biomass, 25–98 vol%) and competitive production costs but require high temperatures and pressures (700–1100 °C, up to 25–33 bar) and substantial capital investment. Microbial fermentation, in contrast, operates under mild conditions (≤60 °C, pH ≤ 7), with lower capital and operational costs, higher energy efficiency (up to 61%), and reduced environmental impact; however, yields are lower, and scalability is limited [105].

Despite these constraints, fermentation is particularly attractive for wet organic wastes and decentralized applications, supporting sustainable waste management and circular bioeconomy goals.

### 5.3. Organic Acids

Organic residues provide sustainable substrates for the microbial production of high-value organic acids, as shown in Table 4. These acids, including lactic, citric, and acetic acids, are widely used in the food, feed, and chemical industries due to their solubility, buffering capacity, and preservative properties [106].

Lactic acid (LA) is essential for producing polylactic acid and several other bio-based chemicals [119]. Its global market value was USD 3.45 billion in 2024 and is projected to reach USD 6.65 billion by 2033 [114]. LA can be obtained from polysaccharide-rich wastes such as restaurant waste, cassava bagasse, and wood hydrolyzate [107,108,109]. For example, *Lactobacillus delbrueckii* subsp. *delbrueckii* CECT 286 produced 51.3 g/L LA from orange peel at 40 °C and pH 5.8, with a yield of 0.9 g/g [120]. Scale-up studies using *Lactobacillus casei* DSM 20011 achieved yields of 78.3% and productivities of 1.12 g/L·h with ricotta whey and pear residues [110].

Citric acid (CA) is another high-demand organic acid, with global production projected to reach 2.91 million tons by 2026 [48]. CA can also be produced through the fermentation of food wastes such as banana peels, brewery waste, apple pomace, pomegranate peel, and cocoa pod husks using *Aspergillus niger* [111,112,113,114].

Acetic acid (AA) is a versatile solvent and chemical raw material, widely used in agriculture, medicine, textiles, adhesives, cosmetics, and food. It also serves as a precursor for polymers such as cellulose acetate and polyvinyl acetate, and functions as an acidity regulator in many chemical processes [121]. Driven by high demand, the global AA market is projected to reach 24.58 million tonnes by 2030. From an industrial perspective, AA is predominantly produced through the biotransformation of ethanol into acetic acid using a mixed culture of acetic acid bacteria (AAB) in specialized bioreactors known as acetators [48]. Industrial acetification, for example, in 100 L acetators, achieves productivities of 1.84 ± 0.03 g/L/h, final AA concentrations of 116.0 ± 0.9 g/L (approximately 3.8% *w*/*v*), and process yields above 94% [122]. In parallel, laboratory-scale studies have explored the production of AA from food and lignocellulosic wastes. For instance, fermentation of kitchen waste with *Lactobacillus plantarum* yielded 35.7 g/L AA, accounting for over 90% of volatile fatty acids [116], while pineapple peels fermented by *Acetobacter pasteurianus* FPB2-3 generated up to 7.2% (*w*/*v*) AA in 16 days [117]. These results highlight that waste-derived AA production is feasible but currently less productive than conventional industrial processes. Lignocellulosic residues such as switchgrass, wheat straw, and sugarcane straw have further supported the production of acetic acid by *Moorella thermoacetica* and *Aurantiochytrium limacinum*, achieving up to 80% of theoretical yields at laboratory scale while reducing media costs by approximately 75% [118].

Overall, microbial conversion of organic residues into LA, CA, and AA demonstrates the potential for sustainable, circular, and value-added chemical production. While laboratory and pilot studies have shown promising results, bridging the gap to commercial-scale application requires systematic optimization of microbial strains, substrates, and bioprocess conditions, alongside economic and life-cycle assessments to ensure industrial viability.

### 5.4. Bioactive Compounds

Fermentation of agri-food residues enhances or releases bioactive compounds, making them valuable for functional foods, nutraceuticals, and dietary supplements. Microbial processing increases the bioavailability of phenolics, flavonoids, and other phytochemicals while supporting circular bioeconomy principles by converting low-value by-products into bioactive compounds, as shown in Table 5.

For instance, fermented pineapple peels treated with *Lactobacillus plantarum*, *Lactobacillus rhamnosus*, and *Aspergillus oryzae* become a rich source of phenolic compounds with elevated antioxidant and anti-inflammatory activity [123]. Likewise, rice bran fermented with *Rhizopus oryzae* becomes a potent source of ferulic acid, increasing to 765 mg/g, along with improved antioxidant properties [124]. Fermentation of soybean dregs by *Neurospora crassa* generates oligosaccharides with prebiotic potential, further illustrating how microbial processes convert residues into functional bioactive sources [125]. Similarly, solid-state fermentation of crude olive pomace with *Aspergillus niger* significantly enhances total phenolic content and antioxidant activity [126]. Similar increases in phenolics, flavonoids, and other bioactive molecules have been reported from fermented pineapple, guava, peanut, and apricot residues using various fungal strains [127,128,129,130,131].

Conventional methods for recovering bioactive compounds from agri-food residues typically rely on solvent extraction and thermal treatments, which are often energy-intensive and can degrade heat-sensitive compounds [132]. In contrast, fermentation, particularly SSF, eliminates the need for solvents, increases yields, and enables efficient recovery of bioactive compounds [133].

### 5.5. Enzymes

Agro-industrial residues also provide low-cost substrates for microbial production of industrially relevant enzymes, including α-amylases, pectinases, lipases, and proteases as shown in Table 6.

Residues such as mango processing waste, wheat bran, and oil cakes serve as substrates for α-amylase production. For instance, *Fusarium solani* produced the highest α-amylase activity from mango waste at pH 4 and 30 °C, with optimal activity at pH 5 and 40 °C [134]. *Aspergillus oryzae* produced α-amylase from low-cost deoiled cakes under SSF, reaching 9868.12 U/gds at laboratory scale and 10,994.74 U/gds at pilot scale in a 600 L fermenter, with good thermal and pH stability, showing potential for industrial use [135]. *Bacillus siamensis* YC-9 also showed secretion of protease, α-amylase, and cellulase when cultivated on rice bran, wheat bran, and corn pericarp [136].

Fruit-derived residues are widely used for pectinase production: for instance, banana peel, coffee pulp, and orange peel supported enzyme synthesis by *Yarrowia phangngaensis*, *Aspergillus* sp., and *Aspergillus cervinus* [137,138,139]. Lipase-producing microorganisms such as *Yarrowia lipolytica*, *Aspergillus terreus*, and *Penicillium simplicissimum* have been cultured on lipid- and protein-rich wastes, including soybean meal, andiroba oil cake, Bati butter, and castor bean biodiesel residues [141,142,143]. Moreover, proteases were generated from wheat bran, soybean meal, cottonseed meal, and orange peel, with the highest activity (262.78 U/g) obtained using a 1:1 mixture of wheat bran and soybean meal by *Aspergillus niger* [140].

Despite these promising results, the commercial application of enzymes derived from agro-industrial residues faces regulatory and safety challenges. Food-grade enzymes must undergo rigorous evaluation for allergenicity, toxicity, dietary exposure, and, in the case of genetically modified strains, additional assessments [144]. Regulatory frameworks vary in the EU; the EFSA requires comprehensive genetic and safety data, whereas the US FDA primarily evaluates the final product under the GRAS (Generally Recognized as Safe) system. Differences in international regulations and the lack of globally harmonized standards can complicate approval and commercialization timelines [145].

Overall, microbial fermentation of organic residues is a versatile and sustainable strategy for transforming low-value waste materials into high-quality proteins, biofuels, organic acids, bioactive compounds, and industrial enzymes. Such processes not only offer economic and environmental benefits but also contribute significantly to waste valorization and the development of a circular bioeconomy in the agri-food sector. While most studies have been conducted at the laboratory scale, further optimization and economic evaluation are needed to fully translate these processes into commercial practice.

## 6. Precision Fermentation—A Biotechnological Tool of the Future

Traditional fermentation is a process known for centuries as a method of food preservation [146]. Precision fermentation is a modern biotechnological tool that evolved from traditional fermentation, combining proven microbiological mechanisms with advances in genetic engineering for the targeted and sustainable production of specific, high-value compounds. This technology is revolutionizing the food system, offering a more efficient and sustainable alternative to traditional fermentation processes [147].

In early 2024, precision fermentation was redefined as “Precision fermentation combines the process of traditional fermentation with the latest advances in biotechnology to efficiently produce a compound of interest, such as a protein, flavor molecule, vitamin, pigment, or fat” [148]. This definition, developed by two key industry organizations, Precision Fermentation Alliance (PFA) and Food Fermentation Europe (FFE), takes into account important details of the fermentation process. First, the intervention occurs at the molecular level. This means that a specific molecular sequence derived from digital databases (rather than directly from animals or plants) is introduced into the microorganism, instructing it to produce a given compound. Second, after fermentation is completed, the produced compound is filtered and separated from the microorganism used. Third, precision fermentation is not a new technology. It has been used for nearly 30 years, for example, in the production of insulin [148], which has become one of the most important and well-known applications of this technology in medicine [149].

**Figure 3:**
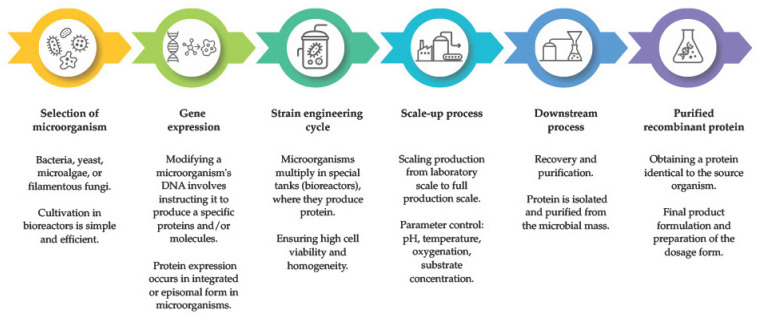
Protein production process using precision fermentation techniques. The diagram illustrates the steps involved in protein production using precision fermentation. Adapted from Knychala et al. [149].

Microorganisms are the basis of precision fermentation because they are the carriers of genetic information and act as the “factory” for producing desired compounds. Their metabolism can be modified to produce specific, desirable compounds. The most commonly used microorganism groups include yeasts (e.g., *Saccharomyces cerevisiae*, *Komagataella phaffii*), bacteria (*Escherichia coli*, *Lactococcus lactis*), molds and filamentous fungi (*Aspergillus niger*, *Trichoderma reesei*), and microalgae (*Chlorella vulgaris*, *Arthrospira platensis*). Microorganisms with GRAS (Generally Recognized As Safe) status are typically selected for programming [146]. An example, simplified process for protein production using precision fermentation is shown in Figure 3.

Precision fermentation is an advanced form of diversification, as it allows microorganisms to be engineered to produce specific molecules using diverse feedstocks and processes, including alternative sources of fermentation feedstock (e.g., food waste) [146]. Globally, there is a lack of harmonization across countries, resulting in scale-up delays, regulatory costs, and legal uncertainty. For example, in the United States, products obtained by precision fermentation may be considered GRAS, based on expert consensus and literature data, or may require a full Food and Drug Administration (FDA) process. In the European Union (EU), food ingredients produced by precision fermentation are covered by several complementary legal instruments. Because there is no uniform legal definition for “precision fermentation” as a separate category of food law, it is assessed as a “novel food.” Regulation (EU) 2015/2283 establishes a regulatory framework for novel foods, defining them as foods not used to a significant degree in the EU before 15 May 1997 [150]. The procedure for authorizing novel foods in the EU is usually time-consuming and relies on detailed documentation on product characteristics, safety, and manufacturing technology [151]. The marketing authorization procedure is handled by the European Food Safety Authority (EFSA). For the use of genetically modified organisms in precision fermentation, Directive 2001/18/EC, Regulation (EC) No 1829/2003, and Commission Regulation (EC) No 641/2004 [152,153,154], as well as regulations on food additives [155] and labeling and traceability requirements [156,157] apply. The legal system is characterized by a strict precautionary approach, which increases the level of consumer protection [151].

**Figure 4:**
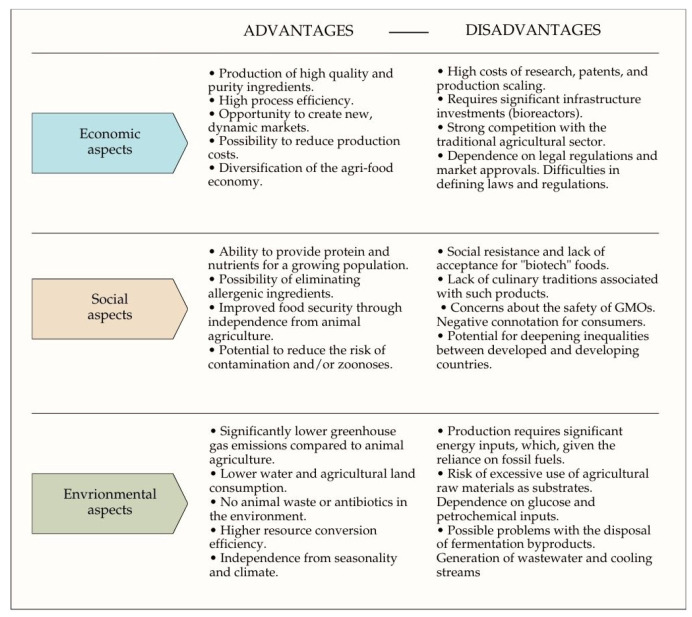
Advantages and disadvantages of precision fermentation. A division into three aspects of life was proposed: economic aspects, social aspects, and environmental aspects. The advantages and disadvantages of using precision fermentation are presented.

In the context of sustainable development, precision fermentation offers numerous benefits (Figure 4). It enables the production of high-quality functional ingredients in a sustainable, efficient, and scalable manner. One of its key advantages is the high process efficiency, allowing for the production of large quantities of pure, homogeneous ingredients with minimal raw material and energy consumption. Compared to traditional methods of animal breeding or plant cultivation, this technology significantly reduces production costs and environmental impact. Furthermore, it enables the diversification of economies and supply chains, creating new opportunities for the food, cosmetics, pharmaceutical, and bioindustrial sectors. It enables the local production of key ingredients, independent of geopolitical conditions. Consequently, precision fermentation is the foundation of a modern bioeconomy [146]. This technology allows for the utilization of resources that theoretically constitute waste, reducing food and feed waste and potentially contributing to the creation of high-value products [146]. Using innovative sources of raw materials for fermentation can facilitate adaptation to the growing popularity of plant-based diets among consumers [18]. More and more consumers are choosing environmentally friendly, sustainably produced, and animal-free products. Furthermore, this type of production is independent of animal production, and at the same time, there is a lower risk of zoonotic diseases, antibiotic resistance, or other contaminants [19]. This type of fermentation is suitable for people with various health problems, such as allergies [18,158]. Environmental aspects are equally important. Precision fermentation reduces emissions and resource consumption. Producing ingredients in bioreactors requires less water, energy, and land consumption, and also reduces greenhouse gas emissions, particularly methane and nitrous oxide [19]. Reducing the need for intensive animal farming can contribute to the protection of biodiversity, reducing deforestation, and preserving natural ecosystems [18]. This technology allows for the production of ingredients independent of climate or soil quality. This is an opportunity for regions where traditional agriculture is difficult [159].

Investing in precision fermentation involves high capital, patent, and operating costs [146,159]. This process requires advanced biotechnological infrastructure, bioreactors, and control systems. Scaling up production is currently not competitive with traditional technologies and requires innovative engineering approaches and optimization of production processes [160]. Scaling up also poses other challenges. Increasing the volume of bioreactors often changes conditions, reducing process efficiency due to changes in the metabolic stability of microorganisms [161]. Precision fermentation requires strictly high-energy and pure culture media, which is associated with high water and energy consumption, while the media components themselves generate high costs [146]. This creates a potential environmental burden associated with a larger-than-expected energy footprint [162]. As mentioned earlier, these processes are subject to rigorous approval processes, and development is delayed by the lack of appropriate legal regulations [151]. Products produced using GMOs often encounter consumer resistance stemming from ignorance and concerns about health safety, interference with the naturalness of food, and trust in the technology. Social barriers can effectively slow down the commercialization of the technology [18,146]. Although the literature often emphasizes the potential of using industrial waste and side streams as sources of carbon and nutrients in precision fermentation processes, in practice most industrial processes still rely on highly purified substrates such as glucose, sucrose, or protein and carbohydrate hydrolysates derived from food raw materials. In practice, this means dependence on food raw materials and potential competition for resources [163].

Precision fermentation technology has significant production potential, but its implementation on an industrial scale requires overcoming numerous technological, economic, environmental, and social barriers. Appropriate process optimization, the development of media based on waste streams and the stabilization of the regulatory and legal framework can bring it closer to the assumptions of sustainable development and economic competitiveness.

## 7. Microbiome and Fermentation as an Element of a Health Strategy

The increasing and unsustainable exploitation of natural resources by modern food production systems poses a serious threat to future food security. This situation highlights the need to introduce high-quality plant-based diets, which can provide an effective solution to current health, environmental, and economic problems [164]. Sustainability in the context of human nutrition can encompass many aspects, such as the impact on climate and natural resources, comparing the carbon footprint and water footprint of plant and animal production, the importance of sustainable dietary patterns, food waste, and the importance of local suppliers and seasonality [165].

The modern Western diet is unfavorable for our microbiome because it is high in calories, low in fiber and nutrients, and rich in highly processed foods high in saturated fatty acids, trans fatty acids, processed meat, artificial food additives, salt, and refined sugar. It is characterized by low diversity and frequent overeating [166,167]. The Western diet is closely linked to increasing environmental degradation, and intensive animal agriculture is associated with the overuse of natural resources (soil degradation, excessive water consumption, eutrophication, the use of vast amounts of agricultural land, deforestation, and biodiversity loss) [168].

Numerous studies on the human microbiome have shown that it plays a key role in nutrition and diet-related diseases [169]. Because the microbiomes of water, soil, plants, and animals play an equally important role in the environment, there has been intensive development of processes to improve the function and composition of the microbiome [165,170]. One aspect that is increasingly being emphasized is the importance of the microbiome in the One Health concept, which recognizes the interdependence of human, animal, and environmental health. The microbiome is a fundamental link in these relationships, playing a role in nutrient cycling, soil quality, crop health, livestock health, and ultimately human health [171]. The modern world faces various challenges. On the one hand, awareness of healthy eating is growing. Plant-based diets are considered more authentic and representative [164]. On the other hand, the effects of environmental degradation caused by industrial food production are increasingly felt. Our dietary choices can support gut microbial diversity, which often aligns with the principles of sustainable development [172]. Diets based on vegetables, fruits, legumes, whole grains, and nuts not only have a real impact on reducing greenhouse gas emissions and water consumption but also provide fiber, which is essential for the proper functioning of the gut microbiome [173,174].

The value of fermented foods in the proper functioning of the human microbiome, and therefore health, is invaluable. It has been proven that a diet rich in fermented foods increases gut microbial diversity (transient or long-term microbial colonization) and reduces inflammatory markers in the body [17]. Fermentation is a combination of tradition and nature [175]. This is crucial because biodiversity is considered a key indicator of metabolic and immune health [176]. Fermentation increases the bioavailability of many nutrients and produces substances with antioxidant, prebiotic, and antimicrobial properties, as well as improving digestibility and extending shelf life [164]. This raises the possibility of using the microbiome to counteract threats to food security [165]. A distinctive feature of fermented products is their locality. Different regions of the world have developed different fermented products, produced using different methods, depending on the location, climate, availability of raw materials, and knowledge, adapted to the culture and culinary customs [167].

In summary, there is a mutual connection and a cause-and-effect chain: “healthy microbiome = healthy people = healthy environment”. Choosing simple, natural, plant-based, and minimally processed foods, and the support you receive for your own health and the environment will be invaluable. The health of the human microbiome affects not only the individual but also the health of populations and the environment. Therefore, its role may be important for achieving the SDGs. Exploring the potential of the microbiome can contribute to the development of innovative and more sustainable strategies for improving public health.

## 8. Postbiotics and Sustainable Development

Postbiotics are a growing field of interdisciplinary research spanning biology, food technology, medicine, and environmental sciences. According to the International Scientific Association for Probiotics and Prebiotics (ISAPP), a postbiotic is “a preparation of inanimate microorganisms and/or their components that confers a health benefit on the host” [177]. The word “postbiotic” comes from the Greek, where “post” means “after” and “bios” means “life.” Other terms for postbiotics include “paraprobiotics,” “parapsychobiotics,” “heat-killed probiotics,” “ghost probiotics,” “ghost probiotics,” “metabiotics,” “tyndalized probiotics,” “bacterial lysates,” “cell lysates,” and “nonviable probiotics” [177,178]. Unlike probiotics, which require the introduction of live microorganisms into the body, postbiotics include inactivated microbial cells, containing or not containing metabolites or cellular components, such as antibacterial peptides, secondary metabolites, organic acids, cell wall components, or exopolysaccharides [177].

The characteristics and application areas of postbiotics are closely related to probiotics, prebiotics, synbiotics, and fermented foods. The synergistic action between these ingredients can increase their bioavailability and effectiveness, which translates into improved health [179]. From a technological perspective, postbiotics are characterized by greater stability and a higher level of safety than the living microorganisms from which they are derived, as their biological activity does not require maintaining viability either during consumption or industrial production [180]. Furthermore, they have a well-characterized chemical composition, which facilitates precise dosage determination [181]. Postbiotics do not affect the sensory properties of food products or disrupt the structure of the food matrix, and their standardization process is relatively simple [182]. Furthermore, they do not carry antibiotic resistance genes, which is significant from a public health perspective [183].

The contribution of the integration of postbiotics into sustainable food systems can be understood in many ways. Postbiotics can be obtained from agri-food industry waste (e.g., whey, plant fibers), unused protein fractions, or fermentation by-products. This approach supports the circular economy, reducing the environmental footprint of production and reducing waste. An important aspect of postbiotics is the ability to produce them in a manner consistent with the principles of the circular economy. Furthermore, the absence of the need to maintain live probiotic cultures reduces the costs associated with refrigeration, transport, and storage stability. Such solutions help reduce food waste, reduce resource consumption, and reduce the environmental footprint [184]. Postbiotics increase the efficiency of fermentation processes. By using microorganisms with high metabolic efficiency, they enable the biotransformation of by-products into valuable bioactive ingredients [185,186]. Postbiotics also exhibit antibacterial and antifungal properties, which can extend the shelf life of products. They also have the ability to reduce bacterial biofilms (Mafe and Buselberg, [187]). In animal husbandry, they help reduce antibiotic consumption, support the immune system and microbiome of production animals, reduce the need for antibiotic therapy, and reduce the risk of resistance [183]. From a safety perspective, postbiotics eliminate the risk of opportunistic infections in susceptible individuals. They play a crucial role in preventive medicine and public health protection. They reduce health and environmental costs, reduce the burden on health systems, limit the use of synthetic drugs, and reduce hospitalizations by supporting the proper functioning of the microbiome [188,189].

As a result, postbiotics can be considered an element integrating food, health, and environmental goals, aligning with global trends in sustainable development and the transformation of food and healthcare systems toward greater efficiency, safety, and ecological responsibility [187].

## 9. Challenges and Limitations of Fermentation-Based Waste Valorization

Fermentation offers a versatile and biologically driven strategy to convert organic residues into valuable products, aligning with the circular economy and sustainable goals. However, scaling up such processes faces a complex array of technical, economic, environmental, and regulatory challenges, which affect both industrial feasibility and the potential environmental and social benefits. Understanding these limitations is essential for designing robust, scalable, and safe processes.

A primary difficulty can lie in the intrinsic heterogeneity and variability of feedstocks. Organic residues from agricultural, industrial, and household sources can vary in nutrient (starch, sugars, protein, and lipid) and moisture content, pH, and impurities such as food particles, metals, or other contaminants, with seasonal and geographical fluctuations further complicating supply chain management [10,190]. Seasonal peaks in fruit/vegetable waste versus continuous production of dairy/brewery by-products affect fermentation kinetics, microbial stability, and yields. Additionally, contamination with pathogens, mycotoxins, or chemical residues imposes stringent quality control measures, particularly for food-grade applications, increasing operational complexity and costs [190,191].

Many residues, especially lignocellulosic materials, require additional pretreatment to release value-added compounds. Mechanical, chemical, or enzymatic pretreatments can improve substrate accessibility but often demand substantial energy and reagents and generate secondary waste streams and toxic byproducts; pretreatment can account for 40% of total process costs in biorefinery processes [192,193,194]. Low-energy alternatives, such as biological pretreatments with microorganisms or enzymes (e.g., white-rot fungi or cellulases), may reduce the environmental impact but may be slower and less predictable at an industrial scale. Inefficient pretreatment limits yield, feasibility, and sustainability. Advances in integrated physical, chemical, and biological pretreatments are expected to improve biomass conversion efficiency, lower energy demands, and support more sustainable renewable-energy and environmental practices [193,195].

Maintaining microbial stability remains one of the major bottlenecks in fermentation-based food biotechnologies. Mixed or spontaneous fermentations using complex substrates often suffer from contamination, metabolic drift, and unpredictable shifts in microbial communities, leading to inconsistent product quality [196,197]. Continuous or open fermentation processes have also been explored as strategies to increase productivity and reduce costs; however, they require careful control of contamination risk and overall process stability, because prolonged cultivations and open conditions increase microbial instability unless feed and product removal are precisely managed [198]. Controlled inoculation improves reliability but increases operational costs and reduces flexibility. Scaling up further amplifies instability due to oxygen and nutrient gradients, shear stress, foaming, and unwanted biofilm formation [196,199]. Biofilm systems can enhance tolerance and productivity, but they introduce additional issues such as slow reactor start-up, carrier clogging, and difficulties in process monitoring and control [199]. Overall, microbial fermentation systems remain hindered by instability, scale-up constraints, monitoring limitations, and regulatory or economic barriers that complicate adoption in sustainable food and biomass valorization [196,197,199]. These challenges underscore the need for interdisciplinary approaches combining microbiome engineering, reactor design, process monitoring, and regulatory compliance to enable reliable and scalable applications.

Downstream processing and product recovery remain major technical and economic bottlenecks in fermentation. Products such as organic acids, bio-based chemicals, and enzymes are often obtained in diluted form and mixed with impurities, requiring energy-intensive separation and purification to meet quality standards [200,201,202,203]. For example, purification of 2,3-butanediol can account for 50–70% of production costs due to its hydrophilicity and complex broth composition [202]. Similarly, starch hydrolysates and other plant-derived bioproducts contain residual colours, proteins, salts, and flavour compounds that must be removed to achieve high-quality outputs [203]. Emerging strategies, such as in situ product recovery, membrane-based separation, liquid–liquid extraction, and adsorption techniques, can improve efficiency and reduce energy consumption, but industrial-scale deployment remains limited [200,201,203]. Overall, although these innovations hold promise, energy demand, process complexity, and variability in feedstock quality continue to limit economic feasibility. Effective downstream strategies are critical to unlock fermentation’s potential for sustainable production.

Logistical and infrastructural issues are also significant. Wet, bulky, and perishable feedstocks necessitate prompt collection, storage, and transport, often requiring cold-chain management or stabilization (drying, freezing, or ensiling), which increases energy consumption and costs [5]. Decentralized micro-biorefineries close to waste generation sites may mitigate some logistical challenges but require investment, local coordination, and new supply-chain models. Lifecycle and environmental considerations must be carefully addressed. While fermentation is generally perceived as environmentally benign, the sustainability benefits depend heavily on process design and system boundaries. Energy and water inputs for pretreatment, sterilization, and downstream purification can offset environmental gains, and biogenic CO_2_ emissions must be accounted for when assessing carbon neutrality [20]. Integrated strategies, including renewable energy use, water recycling, and valorization of residual biomass, are crucial to maximize environmental benefits.

Despite these challenges, ongoing research in metabolic engineering, omics technologies, and process integration is progressively reducing technical barriers. Nonetheless, feedstock variability, process economics, regulatory complexity, and market acceptance remain key constraints. A systemic approach integrating microbiology, process engineering, policy, and socio-economic strategies is required to enable safe, scalable, and sustainable fermentation-based waste valorization.

## 10. Concluding Perspectives

Fermentation offers a versatile, biologically based platform for reclaiming value from organic residues, thereby supporting circularity and achieving SDG targets. To fully realize its potential, research and deployment should focus on:

− Resilient supply chains ensuring consistent feedstock quality,
− Low-energy pretreatment and decentralized processing (e.g., solid-state fermentation),
− Efficient downstream recovery strategies,
− Clear regulatory frameworks for food-grade and feed applications,
− Scaling technology and transferring knowledge to industry, while maintaining process safety and stability,
− Life cycle assessment and environmental impact analysis to ensure real climate and environmental benefits,
− Building social acceptance and consumer awareness of biotechnological value recovery methods,
− Integrating fermentation with other bioprocess technologies within integrated biorefinery production systems.

Cross-disciplinary collaboration among microbiologists, process engineers, supply chain experts, economists, and policymakers will accelerate the translation of lab-scale innovations to industrial deployment. Addressing technical, economic, regulatory, and socio-cultural barriers is essential to establishing safe, economically viable, and environmentally sustainable fermentation-based biorefineries. By integrating scientific innovation with policy support and market awareness, fermentation can evolve from a niche technology to a cornerstone of the global circular bioeconomy.

## Figures and Tables

**Table 1:** Comparison of SSF and SmF for selected microorganisms and products.

Microorganism/Product	Substrate	SSF vs. SmF	Key Observation	Reference
*Neurospora sitophila*– cellulases	Wheat straw	SSF >> SmF	Higher cellulase activity in SSF	[60]
*Aspergillus niger*– cellulases	*Coir waste*	SSF >> SmF	SSF significantly increases cellulase activity	[61]
*Aspergillus brasiliensis*–inulinase/invertase	Agro residues	SSF > SmF	SSF improves enzyme production	[62]
*Aspergillus oryzae*– secreted proteins	Defined medium	Mode-dependent	Different growth/product profiles in SSF vs. SmF	[63]
*Aspergillus niger*–citric acid (industrial)	Sugars/molasses	SmF preferred	Commercial citric acid is mainly produced via SmF	[64]

**Table 2:** Examples of agri-food wastes used as substrates for microbial production of single-cell proteins, including the microorganisms employed and the products obtained.

Source of Waste	Microorganism	Product Obtained	References
Mango, prickly custard apple, pineapple, papaya, banana, mangosteen, cashew apple, cacao, jackfruit, and pomegranate	*Saccharomyces cerevisiae*	Single-cell proteins	[90]
Pineapple waste	*Saccharomyces cerevisiae*	Single-cell proteins	[91]
Orange, banana, sugarcane, garlic, and potato peels	*Aspergillus niger*	Single-cell proteins	[92]
Banana peel, citrus peel, carrot pomace, and potato peel	*Saccharomyces cerevisiae*	Single-cell proteins	[93]
Bread waste	*Rhizopus delemar* CBS 145940	Protein biomass	[94]
Potato protein liquor	*Rhizopus delemar*	Protein biomass	[95]
Coffee wastewater	*Candida sorboxylosa*	Single-cell proteins	[96]

**Table 3:** Examples of agri-food wastes used as substrates for microbial production of biofuels, including the microorganisms employed and the products obtained.

Source of Waste	Microorganism	Product Obtained	References
Leftover bread	*Enterobacter ludwigii*	2,3-butanediol	[98]
Food waste	*Fusarium oxysporum* *Saccharomyces cerevisiae*	Ethanol	[99]
Kitchen waste	*Saccharomyces cerevisiae* and *Pichia stipites*	Ethanol	[100]
Corn by-products	*Clostridium beijerinckii*	Butanol	[101,102]
De-oiled rice bran	*Clostridium acetobutylicum* YM1	Biohydrogen	[103]
Vegetable waste	*Buttiauxella* sp. 4*, Rahnella* sp.* *10 and *Raoultella* sp.* *47	Biohydrogen	[104]

**Table 4:** Examples of agri-food wastes used as substrates for microbial production of lactic, citric, and acetic acids, including the microorganisms employed and the products obtained.

Source of Waste	Microorganism	Product Obtained	References
Cassava bagassa	*Lactobacillus casei* and *Lactobacillus delbrueckii*	Lactic acid	[107]
Restaurant waste	*Streptococcus* sp. *Lactobacillus* sp.	Lactic acid	[108]
Wood hydrolyzate	*Enterococcus faecalis*	Lactic acid	[109]
Orange peel	*Lactobacillus delbrueckii*	Lactic acid	[110]
Ricotta whey and pear residues	*Lactobacillus casei* DSM 20011	Lactic acid	[110]
Pomegranate peel waste	*Aspergillus niger*	Citric acid	[111]
Banana peel	*Aspergillus niger*	Citric acid	[112]
Apple pomace	*Aspergillus niger*	Citric acid	[113]
Brewery wastes	*Aspergillus niger*	Citric acid	[114]
Cocoa pod husks	*Aspergillus niger*	Citric acid	[115]
Kitchen waste	*Lactobacillus plantarum*	Acetic acid	[116]
Pineapple peels	*Acetobacter pasteurianus* FPB2-3	Acetic acid	[117]
Switchgrass, wheat straw, and sugarcane straw	*Moorella thermoacetica* and *Aurantiochytrium limacinum*	Acetic acid	[118]

**Table 5:** Examples of agri-food wastes used as substrates for microbial production of bioactive compounds, including the microorganisms employed and the bioactive products obtained.

Source of Waste	Microorganism	Product Obtained	References
Pineapple peels	*Lactobacillus plantarum*, *Lactobacillus rhamnosus*, and *Aspergillus oryzae*	Phenolic compounds, Antioxidant and anti-inflammatory activity	[123]
Rice bran	*Rizhopus oryzae*	Ferulic acid, gallic acid, and antioxidant activity	[124]
Soybean dregs	*Neurospora crassa*	Prebiotics	[125]
Crude olive pomace	*Aspergillus niger*	Phenolic compounds, antioxidant activity	[126]
Pineapple and guava wastes	*Rhizopus oligosporus*	Phenolic content, antioxidant activity, and antiamylolytic activity	[127]
Peanut press cake	*Aspergillus* *awamori*	Phenolic and antioxidant properties	[128]
Apricot press residues	*Aspergillus niger* and *Rhizopus oligosporus*	Quercetin 3-acetyl-glucoside, chlorogenic acid, neochlorogenic acid, rutin	[129]

**Table 6:** Examples of agri-food wastes used as substrates for microbial production of enzymes, including the microorganisms employed and the enzymes obtained.

Source of Waste	Microorganism	Product Obtained	References
Mango kernel	*Fusarium solani*	α-amylase	[134]
Groundnut, coconut, and sesame oil cakes	*Aspergillus oryzae*	α-amylase	[135]
Corn pericarp, rice bran, and wheat bran	*Bacillus siamensis* YC-9	Protease, *α*-amylase, and cellulase	[136]
Banana peel	*Yarrowia phangngaensis*	Pectinases	[137]
Coffee pulp	*Aspergillus* sp. VTM5	Pectinase	[138]
Peels of orange, banana, carrot, lemon, sweet lime, and apple	*Aspergillus. cervinus* ARS2	Pectinase	[139]
Wheat bran, soybean meal	*Aspergillus niger*	Protease	[140]
Andiroba oil cake and soybean meal	*Yarrowia lipolytica*	Lipase	[141]
Castor bean biodiesel	*Penicillium simplicissimum*	Lipase	[142]

## Data Availability

No new data were created or analyzed in this study. Data sharing is not applicable to this article.

## Abbreviations

The following abbreviations are used in this manuscript:

SDGs	Sustainable Development Goals
GHG	Green-house gas
LAB	Lactic acid bacteria
SmF	Submerged fermentation
SSF	Solid-state fermentation
SCP	Single-cell protein
BDO	2,3-butanediol
LCA	Life cycle assessment
LA	Lactic acid
CA	Citric acid
AA	Acetic acid
PFA	Precision Fermentation Alliance
FFE	Food Fermentation Europe
GRAS	Generally Recognized As Safe
FDA	Food and Drug Administration
EU	European Union
